# Causes of maternal deaths and delays in care: comparison between routine maternal death surveillance and response system and an obstetrician expert panel in Tanzania

**DOI:** 10.1186/s12913-020-05460-7

**Published:** 2020-07-06

**Authors:** Ali Said, Mats Malqvist, Andrea B. Pembe, Siriel Massawe, Claudia Hanson

**Affiliations:** 1 Department of Obstetrics and Gynaecology, Muhimbili University of Health and Allied Sciences, Dar es Salaam, Tanzania; 2 Department of Women’s and Children’s Health, Uppsala University, Uppsala, Sweden; 3 Department of Global Public Health, Karolinska Institutet, Stockholm, Sweden; 4 Department of Disease Control, London School of Hygiene and Tropical Medicine, London, UK

**Keywords:** Maternal mortality, Underlying cause, Medical causes, ICD-MM, Verbal autopsy, Three phases of delays, Maternal death surveillance and response

## Abstract

**Background:**

To reduce maternal mortality Tanzania introduced Maternal Death Surveillance and Response (MDSR) system in 2015 as recommended by World Health Organization (WHO). All health facilities are to notify and review all maternal deaths inorder to recommend quality improvement actions to reduce deaths in future. The system relies on consistent and correct categorization of causes of maternal deaths and three phases of delays. To assess its adequacy we compared the routine MDSR categorization of causes of death and three phases of delays to those assigned by an independent expert panel with additional information from Verbal Autopsy (VA).

**Methods:**

Our cross-sectional study included 109 reviewed maternal deaths from two regions in Tanzania for the year 2018. We abstracted the underlying medical causes of death and the three phases of delays from MDSR system records. We interviewed bereaved families using the standard WHO VA questionnaire. The obstetrician expert panel assigned underlying causes of death based on information from medical files and VA according to International Classification of Disease to Death in Pregnancy Childbirth and Puerperium (ICD-MM). They assigned causes to nine ICD-MM groups and identified the three phases of delays. We used Cohen’s K statistic to compare causes of deaths and delays categorization.

**Results:**

Comparison of underlying causes was done for 99 deaths. While 109 and 84 deaths for expert panel and MDSR respectively were analyzed for delays because of missing data in MDSR system. Expert panel and MDSR system assigned the same underlying causes in 64(64.6%) deaths (K statistic 0.60). Agreement increased in 80 (80.8%) when causes were assigned by ICD-MM groups (K statistic 0.76). The obstetrician expert panel identified phase one delays in 74 (67.9%), phase two in 24 (22.0%) and phase three delays in all 101 (100%) deaths that were assessed for this delay while MDSR system identified delays in 42 (50.0%), 10 (11.9%) and 78 (92.9%).The expert panel found human errors in management in 94 (93.1%) while MDSR system reported in 53 (67.9%) deaths.

**Conclusions:**

MDSR committees performed reasonably well in assigning underlying causes of death. The obstetrician expert panel found more delays than reported in MDSR system indicating difficulties within MDSR teams to critically review deaths.

## Background

In the past decade, maternal mortality has decreased worldwide during the period that the international community was striving to attain Millennium Development Goal 5 [1, 2]. Accelerated and concerted efforts are needed to reach the ambitious Sustainable Development Goal 3 [1, 3]. Currently, the maternal mortality ratio (MMR) in Tanzania is still one of the highest in the world, with most deaths occurring during the intrapartum and immediate postnatal period [4, 5]. To design targeted interventions, data are needed on cause of death as well as underlying factors of three phases of delays. For this purpose the World Health Organization (WHO) has conceptualised the Maternal Death Surveillance and Response (MDSR) system to ensure that local data are available in timely fashion to steer efforts to reduce MMR.

The MDSR system, introduced since 2015 in Tanzania [6], includes identification, notification and review of maternal deaths to stimulate learning from what went wrong. Typically a team of health professionals and local managers review circumstances of deaths, underlying (sometimes called primary medical) causes and contributing factors such as delays in care seeking and provision. The three-delay model provides a conceptual framework to categorize delays in maternal death [7–10]. After completing reviews with analysis and interpretation of data, the team elaborates recommendations for action [11]. The action plans are tailored to address specific underlying medical causes of death and the contributing medical and non-medical factors. To decide on the most adequate strategies, it is important for the MDSR system to record correct and consistent causes of death according to ICD-MM [12].

Medical files are widely used to determine underlying causes of facility maternal deaths. In view of poor documentation of medical files in health facilities [13–15] or in instances when medical records are not available, such as death at home verbal autopsy (VA), is increasingly viewed as an alternative method of standardised interviews with bereaved families [16–18]. Using multiple sources may provide a more complete understanding of the circumstances of death and its causes.

Accurate categorization of causes of death facilitates implementation of recommendations that are specific to prevent maternal deaths and reduce possibilities of under- or overestimation of data. While cause of death assignment by health professionals as part of MDSR reviews is commonly preferred, challenges are reported.

In view of the importance of correct information of cause of deaths as well as contributing factors to inform strategies, we sought to 1) estimate completeness of reporting of facility maternal deaths and 2) compare categorization of medical causes using ICD MM and 3) three phases of delays to maternal deaths between the MDSR system and an expert panel of independent obstetricians. We aimed to identify existing gaps in categorizing correct underlying medical causes and three phases of delays to derive recommendations to improve the MDSR system.

## Methods

### Study design

A cross-sectional study was conducted including 132 maternal deaths from two regions in Tanzania. The deaths had occurred between 1st January and 31st December 2018. Routine MDSR categorization of cause of deaths and the three phases of delay was compared with those assigned by an independent expert panel of obstetricians with additional information from VA. To compute the completeness of maternal deaths reported by the MDSR we used the number of infants that received Bacillus Calmette-Guerin (BCG) vaccine,as a proxy for live births as previously recommended, [19] to calculate the MMR for the two regions in 2018.

### Study setting

The study was conducted in Lindi and Mtwara regions in Southern Tanzania with a total population of about 2 million [20]. The two regions have two regional referral hospitals, 12 district hospitals, four private/mission hospitals, 40 health centres and 399 dispensaries. The MMR in Lindi and Mtwara was 456 and 579 per 100,000 live births in 2013 [21]. The fertility rate is one of the lowest (3.8) in Tanzania. Most women, 80.8% in Lindi and 81.3% in Mtwara give birth in health facilities (dispensary, health centres and hospitals). Caesarean section rates are 6.0% in Lindi and 10.3% in Mtwara [5].

### The MDSR system in Tanzania and categorization of cause of death

Each health facility that provides delivery services in Tanzania has a standard MDSR committee as stipulated in the guideline [6]. In regional and district hospitals, where most deaths occur, MDSR committees are composed of a multidisciplinary team of clinical and non-clinical staff such as obstetricians (if available), medical doctors, clinical officers, nurses and midwives from maternity wards, facility management, laboratory personnel and other supporting staff. The committee meets within 7 days after a suspected maternal death has occurred. Before the meeting, a designated person prepares a narrative summary using information from medical files, interviews of health care providers and relatives who cared for the woman. There is no clear guide on how and which relatives should be interviewed. During the meeting the summary is discussed and when necessary more information is obtained from medical files or health care providers who cared for the woman. Findings from the meeting are summarised in a maternal death reporting form which includes demographic characteristics, medical information, underlying medical cause of death, description of contributing medical and non-medical factors along the three phases of delays and a plan of action [6]. The MDSR guideline recommends the underlying medical cause of death to be categorized following ICD MM rules, but the training and the guideline does not provide a formal training on this. The reporting form in MDSR guideline has a short list of example of causes and ICD 10 codes to be used during reporting. (See Table 6 in Appendix).

### Outcomes

Our main outcome was the underlying medical cause of death defined as disease or condition that started the chain of events that led to death e.g. postpartum haemorrhage (PPH) [12] . Underlying causes of deaths are grouped into nine groups that are mutually exclusive, totally inclusive and descriptive of all underlying causes of maternal deaths. The groups are; 1) Pregnancy with abortive outcome, 2) Hypertensive disorders in pregnancy, childbirth and the puerperium, 3) Obstetric Haemorrhage 4) Pregnancy related infection, 5) Other obstetric complications, 6) Unanticipated complications of management, 7) Non-obstetric complications, 8) Unknown/undetermined and 9) Coincidental causes.

As stipulated in Tanzania MDSR guideline, delays in health care seeking or provision of care deemed to have contributed to the maternal deaths were grouped using the three delays model, stipulating delays 1) to decide to seek care; 2) to reach health facilities for care including transport and 3) to receive appropriate care in facilities [6]. Several delays may contribute to one death. Phase one delays are delays at household and personal level that lead to late or lack of seeking care. It includes the time from the onset of disease at home until the decision to seek care is made by the woman, family or both. Phase two delays are concerned with access to health care such as availability of health facility, roads and transport issues, and constitute time from when the decision to seek care is made until arrival to proper health facility. Phase three delays occur in health facilities and are more concerned with time, equipment and supplies, structure, management errors, human resources and referral system, and constitutes time from admission until adequate treatment or care begins.

### Data sources and measurements

Data collection followed three steps: 1) abstracting information from MDSR documents 2) performing VA and 3) independent obstetrician panel review.

The first author AS, in close collaboration with regional Reproductive and Child Health Coordinators, abstracted information using a pre-defined checklist from maternal deaths narrative summaries, death review report forms and district monthly death report summaries (date of death, age, facility, village and cause of death).

The field team (AS and VA interviewers) then traced families using demographic information such as names of the deceased woman, place of death, district and date of death, home address, name of village/street leader, name of husband/partner and other information, for VA interviews.

Verbal Autopsy interviews were conducted using the translated standard questionnaire provided by WHO [18]. The questionnaire was piloted and the Swahili translation was reviewed and corrected accordingly. In addition to the standard inquiries, questions relating to the three phases of delay were added.

The field team commenced the process of finding families for VA interviews by visiting and enquiring in the facility where death occurred or where the deceased woman attended antenatal clinic. They were then taken to the family through local government leaders. At the family’s home, after being introduced they explained in detail the purpose of VA. Then one of the interviewers identified person (s) that was (were) present during illness and death and conducted VA with them.

Using the coded VA questionnaires as well as copies of available medical files a group of experts, consisting of three experienced obstetricians in MDSR reviewed all maternal deaths. Two of them were from Muhimbili University of Health and Allied Sciences and had never worked in the regions and one was from Mtwara regional hospital. The latter was included to help the panel understand the context better especially information in VA. The author, AS, was among the panel members and had previously been trained on using ICD-MM. All the three panel members neither conducted the VA interviews nor documented any information from the reviews.

The three panel members reviewed all the deaths together by reading through the information in VA questionnaire and available medical files. Then they discussed the findings and made their decision by consensus. The cause of death was agreed if at least two of the panel members said the same cause of death. First, the expert panel went through VA questionnaires and determined the underlying cause from the information by consensus. Second, the panel went through the medical files and reviewed all available information. Based on these two sources, the panel determined the 1) underlying cause of death including the ICD coding, 2) contributing medical causes and 3) three phases of delays, all by consensus [12]. The three panel members reached consensus in all deaths that were reviewed even though there was a plan to consult another obstetrician in case of no agreement. This was never used since there was consensus in all deaths.

### Quantitative variables

Data were processed using MS Excel and then transferred to SPSS computer program version 25. Proportions of each underlying medical cause categorized by MDSR system and the expert panel of obstetricians were computed. Underlying medical causes and differences between the routine MDSR system and obstetricians panel were tabulated. As the routine MDSR system used a shortlist of ICD codes while the expert panel used the full number of ICD-MM codes and groups, comparison had to use a pragmatic approach. For example, when the obstetricians panel categorized a death to be caused by PPH due to atony, coagulopathy or retained placenta, this was considered to be in agreement if MDSR system categorized the same death as PPH (non traumatic). Also PPH (traumatic) for MDSR system was decided to be in agreement if obstetricians’ panel categorized the same case as PPH (vaginal tear, cervical tear, extension of uterine incision during caesarean section).

### Statistical methods

Cohen’s K statistics were used to determine the level of agreement in categorizing the underlying causes and the three phases of delays. We defined < 0 as no agreement, 0–0.2 as slight agreement, 0.21–0.4 as fair, 0.41–0.6 as moderate, 0.61–0.8 as substantial and 0.81–1 as almost perfect agreement [22].

## Results

**Fig. 1:**
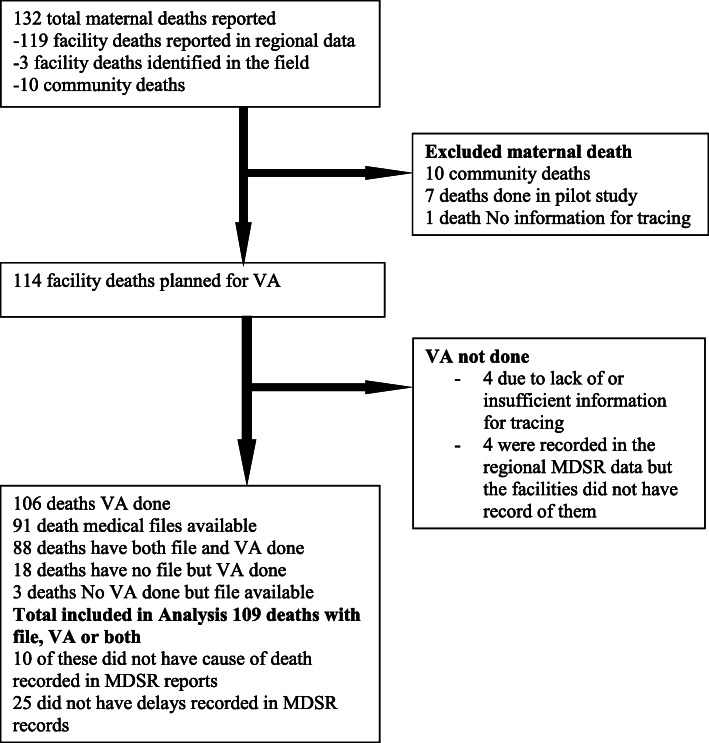
Flow chart of maternal deaths included in the study. Our final analysis included 109 deaths. VA was performed for 106(92.9%) deaths and medical files of 91(83.5%) women could be traced. Piloting our approach was done based on seven maternal deaths which were later excluded from analysis. Of the 132 deaths, 10 were community deaths and no clinical records were available. The recording of one death was so minimal that no information to trace the family was available. Three facility deaths were identified in the field in which two of them were identified during visits to the community and one was reported by the district health office but not reported by the routine regional MDSR system. Out of the 8 deaths that could not be traced for VA, 4 were because the demographic information was not sufficient to trace the family in the villages. The other 4 deaths were reported in the regional MDSR data but, there was no record in facility data. It was later revealed that these were suspected maternal deaths that were reported anonymously to the region but the regional office did not follow them up to confirm whether they were maternal deaths (Fig. 1)

In the year 2018, a total of 132 maternal deaths were reported in the study regions. According to District Health Information System, the total number of children that received BCG vaccine (as a proxy for live-births) for that year in the two regions was 96,265. Thus according to the MDSR system, MMR was 137 per 100,000 live births with 95% Confidence Interval of 115 to 163 deaths per 100,000 live births. Our final analysis included 109 deaths (Fig. 1). Comparison of causes of death was done for 99 deaths while delays were analysed in 109 and 84 deaths for expert panel and MDSR respectively.

More than half 65(59.6%) of women who died were ≥ 30 years (median 31 years and Inter-quartile range of 25–36), 64(58.7%) had primary education, 76(69.7%) were married/living with partner and 69(63.3%) were peasants. Most 52(47.7%) women were sick for less than a day and 56 (51.4%) died within 24 h of delivery. More than three quarters died in the postpartum period, more than half 65 (70.6%) had a live-born baby before dying and 56 (60.2%) gave birth by caesarean section, 49 (45%) of deaths were reported in district hospitals and 20 (18.3%) in regional hospitals (Table 1).

**Table 1:** Demographic and medical characteristics of the Maternal Deaths (*N* = 109)

Demographic and medical characteristics	Frequency	Percent
Age groups		
< 20	10	9.2
20–29	34	31.2
30–39	54	49.5
40 and above	11	10.1
Median age	31	
IQ range	25–36	
Education level^a^		
No formal education	23	21.1
Primary education	64	58.7
Secondary education	15	13.8
Higher education	4	3.7
Occupation^a^		
Unemployed	3	2.8
Employed	7	6.4
House wife	15	13.8
Self employed	4	3.7
Petty trader	9	8.3
Peasant	69	63.3
Marital Status^a^		
Single/Divorced	31	28.4
Married/cohabiting	76	69.7
Duration of sickness before death (days)^a^		
< 1 day	52	47.7
1–7	41	37.6
8–14	10	9.2
> 14	3	2.8
Place of delivery /abortion^b^		
Hospital	74	67.9
Health centre	14	12.8
Dispensary	2	1.8
Home	5	4.6
On the way to facility	2	1.8
Type of facility reporting death		
Regional hospital	20	18.3
District hospital	49	45.0
Mission hospitals	26	23.9
Health centre	12	11.0
Dispensary	2	1.8
Timing of death		
Antepartum	10	9.2
Intrapartum	9	8.3
Postpartum	90	82.6
Died within 24 h of delivery/abortion		
Yes	56	51.4
No	34	31.2
Died before delivery	19	17.4
Delivered live baby^b^		
Yes	65	70.6
No	27	29.4
Mode of delivery^b^		
Spontaneous Vaginal Delivery	36	39.2
Caesarean Section	56	60.2

^a^3 Maternal Deaths had no information available and VA was not done

^b^12 died with baby in uterus and baby never delivered and 5 abortion/ectopic

Traumatic and non-traumatic PPH was the most common cause of death categorized by both groups. Obstetricians panel and MDSR system categorized the same underlying causes in 64/99 (64.6%) maternal deaths (K statistic 0.60, moderate agreement) (Table 2).

**Table 2:** Categorization of underlying medical causes and ICD codes by obstetrician experts and MDSR system (*N* = 99)

Underlying medical cause of death	Obstetricians	Obstetricians ICD codes	MDSR	Both
Eclampsia	19	O15.0,O15.1,O15.2	15	14
PPH (non-traumatic)	18	O72.0, O72.1,O72.3	15	12
PPH (traumatic)	8	O71.3,O71.4,O71.8,O71.9	11	6
PPH	6	O72	8	5
High spinal anaesthesia	7	O74.2	6	5
Puerperal Sepsis	6	O85	7	5
Ruptured uterus	7	O71.1	2	1
Unsafe abortion	3	O05.0	2	2
Severe Anaemia	3	O99.0	4	3
Peripartum Cardiomyopathy	4	O90.3	2	2
Ectopic Pregnancy	2	O00	2	2
Obstetric embolism	2	O88	3	1
Severe Preeclampsia	2	O14.1	1	1
Burn Wounds	1	T22	1	1
Heart Disease	1	I05.9	1	1
Septic abortion	1	O03.0	1	1
Severe Pneumonia	1	J15.8	1	1
Pneumocyctic jirovecii Pneumonia	1	B20.6	1	1
Obstructed labour	0	O65	4	0
*Others	7	O71.5,B45.1,B50.8, O45.0, O03.1	12	0
Total	99		99	64

*Others Obstetricians: (Meningitis, Severe malaria, Undetermined, Abruptio placenta, Incomplete abortion, bladder injury)

*Others MDSR (Brain hypoxia, haemorrhagic shock, Congestive cardiac failure, HELLP syndrome, Intracerebral haemorrhage and postural hypotension)

The obstetricians’ panel categorized 21 deaths as caused by hypertensive disorders in pregnancy, childbirth and puerperium and 15 agreements occurred with the MDSR system. The MDSR system categorized two of these deaths in group 3 (obstetric Haemorrhage), two in group 4 (pregnancy-related infection), one in group 5 (other obstetric complications) and one in group 7 (non-obstetric complications). Overall, out of 99 deaths both obstetricians` panel and MDSR system categorised 80(80.8%) in the same ICD-MM group (K statistic 0.76, substantial agreement) (Table 3).

**Table 3:** Level of agreement of the ICD-MM groups between obstetricians panel and MDSR system (*N* = 99)

ICD MM Groups From MDSR System									
ICD MM Groups From Experts	1.Pregnancy with abortive outcome	2.Hypertensive disorders in pregnancy	3.Obstetric Haemorrhage	4.Pregnancy related infection	5.Other Obstetric complications	6.Unanticipated Complication Management	7.Non obstetric complications	8.Unknown/undetermined	9.Coincidental causes
1.Pregnancy with abortive outcome	**6**	0	1	0	0	0	0	0	0
2.Hypertensive disorders in pregnancy	0	**15**	2	2	1	0	1	0	0
3.Obstetric Haemorrhage	1	1	**35**	0	2	0	0	0	0
4.Pregnancy related infection	0	0	0	**5**	1	0	0	0	0
5.Other Obstetric complications	0	0	0	1	**6**	0	1	0	0
6.Unanticipated Complications of Management	0	0	0	0	0	**5**	1	1	0
7.Non obstetric complication	0	0	0	0	0	0	**7**	0	0
8.Unknown/undetermined	0	0	0	0	1	1	1	**0**	0
9.Coincidental causes	0	0	0	0	0	0	0	0	**1**

The obstetricians’ panel identified more delays in all three categories than the MDSR system There was high percentage agreement in identification of phase-three delays 73 (86.9%) while there was slight agreement in specifying phase-two and phase one delays (K statistic 0.2) (Table 4).

**Table 4:** Comparison of identification of three delays to maternal deaths between obstetrician experts and MDSR system

Phases of delays	Obstetricians (***N*** = 109)	MDSR systems (***N*** = 84)^**b**^	Agreement (%)	K statistic
	Frequency (%)	Frequency (%)		
Phase one delay	74(67.9)	42(50.0)	32(38.1)	0.2
Phase two delay	24(22.0)	10(11.9)	4(4.8)	0.2
Phase three delay^a^	101(100)	78(92.9)	73(86.9)	Not calculated

^a^Obstetricians’ panel could not identify delays for 8 maternal deaths in health facilities because there were no medical files and VA was not informative about third delays. Also K statistic was not calculated due to presence of delays in all cases reviewed by MDSR

^b^Missing information of delays identified in MDSR system for 25 maternal deaths

“Delays in decision-making” was the predominant phase-one delay according to the obstetricians’ panel 57 (77.0%) and MDSR system 23 (54.8%). In phase two delays, MDSR system identified more 6 (60.0%) “Delayed arrival to health facility” than obstetricians’ panel 10 (41.7%). The obstetricians’ panel indicated that human errors and mismanagement was assessed to occur in 94 (93.1%) of maternal deaths as compared to 53 (67.9%) by the MDSR system. Agreement in identifying delays ranged from no agreement to slight in most of the categories. There was moderate agreement (K statistic 0.41) in identifying delayed referrals (Table 5).

**Table 5:** Comparison of identified delays to maternal deaths between obstetricians’ panel and MDSR system

	Obstetricians	MDSR system	
	Frequency (%)	Frequency (%)	
**Phase one delays**	***N*** **= 74**	***N*** **= 42**	**K statistic**
Delay in decision making	57(77.0)	23(54.8)	0.04
Delayed referral from home	40(54.1)	17(30.5)	0.30
Failure to recognize problem	25 (33.8)	16(38.1)	0.24
Unwillingness to seek care	15(20.3)	6(14.3)	0.30
Traditional practices	4(5.4)	4(9.5)	0.05
Poverty	2(2.7)	1(2.4)	0.00
Delay in starting antenatal care	17(23.0)	10(23.8)	0.23
**Phase two delays**	***N*** **= 24**	***N*** **= 10**	
Delayed arrival to health facility	10(41.7)	6(60.0)	−0.5
Lack of money for transport	10(41.7)	2(20.0)	0.00
Lack of transport from home	10(41.7)	1(10.0)	−0.33
No facility within reasonable distance	4(16.7)	1(10.0)	0.00
Bad roads	2(8.3)	0(0.0)	0.00
**Phase three delays**	***N*** **= 101**	***N*** **= 78**	
Human errors or mismanagement	94(93.1)	53(67.9)	0.16
Delayed management after admission	77(76.2)	30(38.5)	0.22
Inadequate skills of the provider	64(63.4)	44(56.4)	0.16
Delayed arrival from referring facility	44(43.6)	21(26.9)	0.41
Suboptimal antenatal care	37(36.6)	26(33.3)	0.05
Lack of supplies and equipment	10(9.9)	34(43.6)	0.13

## Discussion

### Main findings

We report a moderate agreement of the categorization of underlying causes of maternal deaths assigned by the standard MDSR committee compared with those assigned by an independent expert panel of obstetricians supported with additional information from VAs. Both groups assigned the same underlying medical cause in 64.6% of deaths. Substantial agreement (K statistic 0.76) was found when ICD-MM groups were compared.

Phase-one delays were identified in 68%, phase-two in 22% and phase-three in 100% by the obstetrician panel as compared to delays in 50% (1st delay), 12% (2^nd^delay) and 93% (3rd delay) identified by the MDSR system. The obstetrician panel found that human errors or mismanagement had occurred in 93.1% of deaths while the MDSR system reported this in 67.9% of deaths with moderate agreement in identifying delays in referral system. The MMR in the two regions was estimated at 137 per 100,000 live births.

### Assigning underlying cause

Both, the obstetricians’ panel and the MDSR system assigned PPH as the most common underlying cause of death. Overall there was substantial agreement in categorizing the underlying cause of death and the ICD-MM group of causes with high K statistic of 0.76. This is in contrast with other studies in Sub Saharan Africa and the US, that have shown significant differences when researcher- assigned causes of death were compared with health care providers’ [23–26]. The substantial agreement in our study could be due to the national training of the MDSR committees on the use an ICD10 shortlist and orientation on ICD 10 codes.

Although most deaths were categorized similarly, the routine MDSR system still reported some contributing causes such as hypoxia, intracerebral hemorrhage, and hemorrhagic shock as underlying causes. This also has been observed in Kenya, Malawi, Nigeria, South Africa and Zimbabwe where contributing or immediate causes were indicated as the underlying causes [25]. This highlights the importance of training providers of the definition of underlying cause of death. This definition can be used differently on the same death depending on the setting as it was the case in a study done in UK and the Netherlands. The study found the UK Confidential Enquiry into Maternal Deaths and the Dutch Audit Committee Maternal Mortality and Morbidity categorized different underlying cause for the same death due to differences in interpretation of the underlying cause and the health system approach to prevention of death [27].

### Identifying the three delays

The obstetricians’ panel identified more delays in phase one (68%) and phase three (100%) compared to the MDSR system. It is of note that the MDSR system indicated no phase-three delays (provider factors) in six maternal deaths while the obstetricians’ panel identified phase-three delays in all deaths. It was not clear whether the MDSR system could not identify delays or efforts tried to shift blame to the deceased women. A culture of blame is one of the major obstacles in maternal death reviews [28, 29]. The obstetricians’ panel indicated human errors in management, substandard decision-making, mismanagement and poor skills in performing medical procedures. These may have led to complications and ultimately to death. In contrast, the routine MDSR system was less inclined to indicate such errors and did not report errors in management in most cases that led to death. Other studies have proposed that most maternal deaths occur due to delays in medical care while delays in seeking care are less important [23, 30–33]. Since Tanzania is seeing increased facility utilization and facility births, it is imperative for health care providers to identify substandard care that can be addressed to save lives [5].

### Maternal death data

The two regions reported a total of 122 facility- and 10 community-related maternal deaths for the year 2018. This corresponds to a MMR of 137 per 100,000 live births, which falls short of most recent national and international estimates of 556/100,000 and 524/100,000 respectively [4, 5]. It points to under-reporting of maternal deaths most likely due to missed deaths both in the facility and community.

Underreporting of deaths was also revealed during our field activities as we identified three facility deaths that were not reported to higher levels of the health care system. Furthermore, four deaths had no records in facilities where they were reported to have taken place and had not been reviewed. This shows existing inconsistencies in identification, notification and reporting of maternal deaths in facilities. The problem of underreporting of maternal deaths is universal all over the world even in countries with well-developed vital registration systems. Studies in the United States, France, Taiwan and Netherlands have all revealed underreporting of maternal death in the health system [34–37]. The situation is also true in low and middle income countries where there is missing or late notification and reporting of maternal deaths in health system [38, 39]. The main reasons for underreporting reported in both high and low income countries are misclassifications, missing death in early pregnancy and abortion, missing indirect deaths, incomplete feeling of death certificates and missing deaths outside facilities.

### Strength and limitations

The main strength of this study is the use of an expert panel consisting of experienced obstetricians who have knowledge of both ICD-MM and MDSR. All three obstetricians received explicit training and have taken part in MDSR in their home institutions. The study used data previously documented, thus the committees did not have prior knowledge of the study which removed social desirability bias if data collection would have been done prospectively.

The expert panel was supported with additional information from the VA. Using both VA and medical files ensured that the expert panel had enough information to identify underlying causes and delays. The fact the VA was used, and thus community factors were explicitly investigated, could possibly explain more delays in first phase identified by the expert panel. However, it is also difficult to know how much information was sought from the family by the MDSR committees. The committees are required to seek information from family members even though there is no formal tool such as VA questionnaire to guide that process.

This study adds to the body of evidence of the reliability of cause of deaths assignment by MDSR teams. Most other studies have assessed how well the providers categorize the underlying medical causes of death in comparison to an external expert panel or computer programs using ICD 10, thus two other systems with major limitations. Our strategy to enhance the work of the expert committee with VA is likely to have led to a more robust assignment of the cause of deaths, thus comparing with a likely gold standard.

Main limitation is the fact that there were multiple MDSR committees that reviewed the deaths. This could pose a problem for Cohen’s K statistic which is recommended for comparing two raters. But each death was reviewed by one MDSR committee and one panel, indicating a two-way comparison for each case. Also, the nature of the study led to missing of some data due to lost documentation, but VA helped in filling information gap in some of these cases.

Another limitation is that only facility deaths were included. While community deaths ought to be reported, the MDSR system is not clear on how these deaths are to be reviewed. In addition, there are no available summaries, review information or any other information about circumstances that would have been needed to include these deaths in our study. It is important to note that the distribution of causes of deaths is not representative for the total population in the two regions.

## Conclusions

The MDSR system performed reasonably well in assigning the underlying cause of deaths according to ICD-MM in contrast to what other studies have indicated. The obstetricians’ panel found more delays of care provision than reported in the MDSR system, indicating difficulties within MDSR committees to critically review maternal deaths. The committee members should have training and support in identifying lessons to be learned in facilities and avoid shifting of blame to the family and deceased.

## Data Availability

Data sets used and/or analysed during the current study are available from the first author on request.

## Appendix

**Table 6:** MDSR Maternal death reporting form. This is the form used to report maternal death information in the MDSR system after review

1. Name of Reporting Health Facility __________________	2. Facility unique ID number (YYYY/Number) ________________	3. Address of the deceased		
		Ward	______________	
		Division ____________________		
		District	______________	
		Region	______________	
**Deceased Information**				
4. Date of Death (DD/MM/YYYY) ____/____/_____	5. Age at death: ___ Years		6. Gravidity ________	
7. Parity __________	8. Marital status **(circle what applies. Only one response allowed)**			
	1. Married		4. Cohabiting	
	2. Single		5. Separated	
	3.Widowed		6. Divorced	
9. Level of education **(circle whatapplies)**	1. None		4. Higher education	
	2. Primary		5. Unknown	
	3.Secondary			
10. Occupation **_________________________**	11. Admission at the health facility			
	Date (DD/MM/YY) **__________________**		Time ______________	
**Antenatal Care (ANC)**				
12. Attended ANC?	1. Yes	2. No	3. Not known	
13. Where was the ANC done?	1. Dispensary		4. Other (specify) _________	
	2. Health centre		5. Had not attended yet	
	3. Hospital			
14. Number of ANC visits			Not applicable (Had not attended yet)	
15. Basic package of services provided on ANC **(Circle what applies)**	Syphilis screening		1. Yes 2. No. 3. Unknown	
	Hgb,		1. Yes 2. No 3. Unknown	
	HIV status		1. Yes 2. No 3. Unknown	
	Blood group		1. Yes 2. No 3. Unknown	
	BP measurement during the follow up		1. Yes 2. No 3. Unknown	
	Urinalysis		1. Yes 2. No 3. Unknown	
	Fe/FoL supplementation		1. Yes 2. No 3. Unknown	
	TT immunization		1. Yes 2. No 3. Unknown	
16. Diagnosis on admission **(circlewhat is appropriate)**	1.Normal labour		10. Ectopic pregnancy	
	2. Eclampsia		11. Previous C/S scar	
	3. Hypertensive disorders without eclampsia			
	4. Nursing mother		12. Violence	
	5. HIV/AIDS		13. Obstructed labour	
	6. Antepartum haemorrhage		14. Severe malaria	
	7. Postpartum haemorrhage		15. Ruptured uterus	
	8. Incomplete abortion		16. Anaemia	
	9.Sepsis		17. IUFD	
			18. Others (Specify) … … …	
17. Name and Place of Delivery/abortion **(circle what applies)**	1. Hospital		5. Delivery before arrival	
	2. Health centre		6. Home	
	3. Dispensary		7. Not applicable (in case undelivered	
	4. Maternity home			
18. Date of death (DD/MM/YYY) ______________________	18 b. Place of Death **(circle what applies)**			
	1. at home		4. at Hospital	
	2. at dispensary		5.on transit to facility	
	3. at health centre		6. Other specify	
19. Duration from onset of complication to time of death			20. When did death occur?	
	_________(hours/days)			
			1. Before Intervention	2. During intervention
21. Timing in relation to pregnancy	1 = Antepartum		2 = Intrapartum	3 = Postpartum
**Delivery and related information**				
22. Mode of delivery	1. Spontaneous vertex delivery		6.Laparotomy/Hysterotomy	
	2. Emergency C/S		7. Other … … ………………… … ..	
	3. Elective C/S		8. Not applicable (had not delivered yet)	
	4. Vacuum extraction			
	5. Breech delivery			
23. Delivery attendant	1. Nurse/midwife		6. Assistant Clinical officer	
	2. Medical Officer		7. Traditional birth attendant	
	3. Obstetrician		8. Other______________________	
	4. AMO		9. Not applicable (had not delivered)	
	5. Clinical officer			
24. In case of caesarean section/laparotomy/Hysterotomy (fill in or circle what applies)	1. Indication of surgery _____________________________________________			
	2. Duration of surgery: a. 1 h or less b. More than 1 h			
	3. Type of anaesthesia used: a. General b. Spinal c. Not recorded			
	4. Time from decision to performing surgery …… .hrs … … ...mins			
	5. Not a C-section/laparotomy			
25 Pregnancy outcome **(circle what applies)**	1.Live baby		2. Fresh still birth	
	3. Macerated stillbirth		4. Ectopic	
	5. Abortion			
26. Was a post mortem done?	1 = Yes		2 = No	
	What was the diagnosis?			
**Cause of death**				
27. Direct cause **(Circle what applies. Only one choice allowed)**	• O0 Ectopic pregnancy • O14.1 Severe pre eclampsia • O15 Eclampsia • O85 Puerperal sepsis • O64 Obstructed labour-Malposition/Malpresentation • O65 Obstructed labour-Maternal pelvic abnormality • O66 Obstructed labour-Other causes • O44.1 Placenta praevia • O45.0 Abrutpio placentae • O71 PPH- Trauma • O72 PPH- Non traumatic • O08 Abortion • O74 Anaesthetic complication • O88 Embolism			
28. Indirect cause	• O99.0 Anaemia • O98.6 Malaria • O98.7 HIV and AIDS • O93.3 Cardiomyopathy • T65 Herbal intoxication • O24 Diabetes Mellitus • O98.0 TB • Others Specify...............			
29. Other causes	• O95 Unspecified or unknown cause of death			
30. Underlying medical conditions that could have contributed to the death	______________________________________________			
	______________________________________________			
**31. Contributory factors and non-medical causes of death (Tick all that apply)**				
Delay 1	Traditional practices	Lack of decision to go to health facility		
	Family poverty	Unwillingness to seek medical help		
	Failure of recognition of the problem	Delayed referral from home		
	Delay in starting antenatal care			
Delay 2	Delayed arrival to referred facility	Lack of transportation		
	Lack of roads	No facility within reasonable distance		
	Lack of money for transport			
Delay 3	Sub optimal antenatal care			
	Delayed arrival to next facility from another facility on referral			
	Delayed or lacking supplies and equipment (specify) _______________________			
	Delayed management after admission			
	Human error or mismanagement (specify) ____________________________			
	Inadequate skills of provider (specify) _______________________________			
Others	(specify) ___________________________________________________________________			
32. Could this death have been avoided?	Yes	No		
	Comment_________________________________________________ __________________________________________			
33. List the avoidable factors, missed opportunities or substandard care – why did this happen?				
34. Summarize the case				

## Abbreviations

ICD-MM: International Classification of Disease on Death in Pregnancy, Childbirth and Puerperium
MDSR: Maternal Death Surveillance and Response
MMR: Maternal Mortality Ratio
PPH: Postpartum Hemorrhage
VA: Verbal Autopsy
WHO: World Health Organization
